# Bioregulatory event extraction using large language models: a case study of rice literature

**DOI:** 10.1186/s44342-024-00022-3

**Published:** 2024-10-31

**Authors:** Xinzhi Yao, Zhihan He, Jingbo Xia

**Affiliations:** College of Informatics, Hubei Key Laboratory of Agricultural Bioinformatics, Huazhong Agricultural University, Wuhan, 430070 China

**Keywords:** Oryza sativa, Bioregulatory event, Text mining, Large language model, Prompt engineering

## Abstract

The extraction of biological regulation events has been a key focus in the field of biomedical nature language processing (BioNLP). However, existing methods often encounter challenges such as cascading errors in text mining pipelines and limitations in topic coverage from the selected corpus. Fortunately, the emergence of large language models (LLMs) presents a potential solution due to their robust semantic understanding and extensive knowledge base. To explore this potential, our project at the Biomedical Linked Annotation Hackathon 8 (BLAH 8) investigates the feasibility of using LLMs to extract biological regulation events. Our findings, based on the analysis of rice literature, demonstrate the promising performance of LLMs in this task, while also highlighting several concerns that must be addressed in future LLM-based application in low-resource topic.

## Introduction

The complex interactions between genes and phenotypes within organisms have long been a central issue in biological research, resulting in a vast body of scientific literature. Within the biomedical natural language processing (BioNLP) community, the extraction of this type of knowledge is commonly referred to as bioregulatory event extraction. For this purpose, several corpora have been developed. For instance, the GENIA corpus [1, 2] utilizes nine types of events, including regulation, positive regulation, and negative regulation, which capture the bioregulatory processes in the literature. Similarly, the pathway curation corpus [3] and cancer genetics [4] corpus focus on elucidating reaction pathways and genetic pathology in organisms. Moreover, the active gene annotation corpus (AGAC) [5–7] defines genetic alteration-caused regulatory events (GARE) to capture the role of molecular-level regulation of genetic alterations in organisms.

However, existing NLP methods face two major challenges in the extraction of biological events. Firstly, the traditional multi-step text mining pipeline often leads to cascading errors. Secondly, the choice of corpus limits the scope of subject coverage, necessitating more resources for specific research areas, such as plant biology. Joint models, which have emerged in recent years, can alleviate the cascading errors brought by the pipeline methods, but they often require training a large number of parameters from scratch, thus also demanding a large-scale training set [8]. Today, LLM has demonstrated powerful semantic understanding and extensive knowledge background in broad range of NLP tasks, such as named entity recognition (NER), named entity normalization (NEN), and relation extraction (RE). Leveraging strong capacity of LLM in semantic comprehension, prompt-based LLM application opens avenues to alleviate the cascading errors inherent in text mining pipelines and handle the above challenges. In this work, presented during the 8th Biomedical Linked Annotation Hackathon (BLAH8), we leveraged a large language model Kimi from Moonshot AI, for bioregulatory event extraction. Kimi is an AI assistant developed by Moonshot AI, specializing in processing and understanding extensive texts with its advanced language model capabilities. It offers a range of features from document summarization to coding assistance, designed to enhance productivity and efficiency for users across various fields. Specifically, we adopted the definition of GARE and attempted to use LLMs to generate structured GARE directly from raw text. We select rice literature as a representative for low-resource texts, while the limited pre-trained data in this field enables a specific exploration of the capacity of LLM in knowledge curation in the low-resource domain.

Through the evaluation of the rice GARE extraction task, we compared the results between the LLM-based method and the conventional deep-learning based pipeline. Finally, the LLM-based method reaches comparable F-score, higher precision, and lower recall. We also highlight several concerns in LLM applications, including the challenge of LLM application in a low-resource domain, the computational cost, and the evaluation criteria for prompt design.

## Methods

In this section, we outline the preliminary preparation of this project and detail the experiments conducted during the BLAH 8.

## Annotated rice corpus

In the preliminary preparation of this project, we utilize a pre-developed rice-GARE annotation corpus. This corpus serves as both the prompt samples provided to LLM as well as the golden dataset for outcome evaluation. In this corpus, following the knowledge pattern ruled by AGAC [7], GARE is defined as a genetic alteration (e.g., overexpression) that occurs in a gene (e.g., OsZHD2) and affects the downstream biological process or trait (e.g., root growth, meristem activity) through a trigger word (e.g., improve, enhancing).

**Fig. 1:**
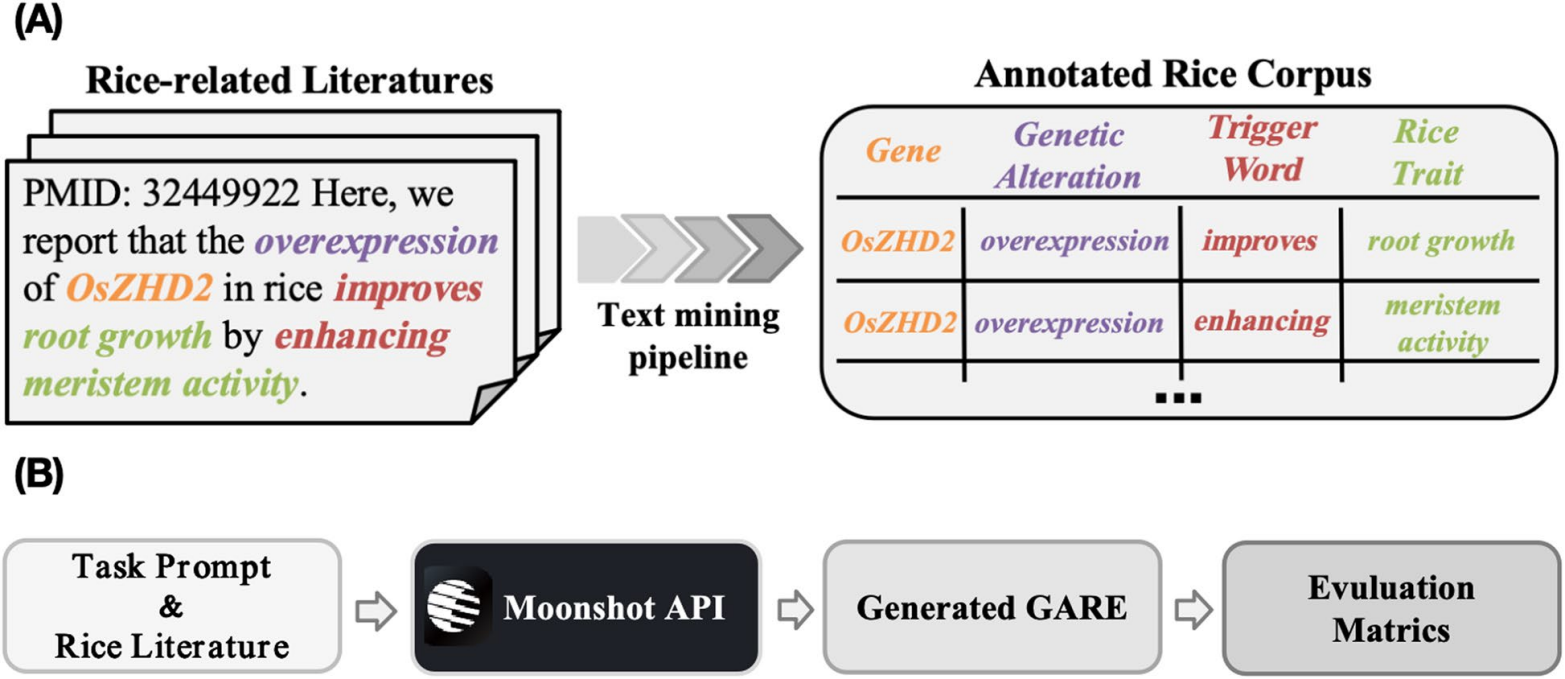
Preliminary preparation and workflow of the project. **A** Annotated rice corpus construction relies on a multi-step text mining pipeline. **B** Flowchart of the LLM for rice-GARE generation and evaluation

As shown in Fig. 1A, we collect the rice-related literature from PubMed and PubMed Central. Then, a four-step AGAC-based text-mining pipeline is employed to extract the GARE from the literature, including NER, NEN, RE, and rule-base GARE extraction, resulting in the structured annotated rice corpus. Specifically, the NER and RE steps in pipeline rely on the BioBERT model, a BERT model pre-trained on biomedical texts, with additional Conditional Random Field (CRF) decision layers and Softmax classification layers for sequence labeling and relation classification tasks, respectively. The model are further fine-tuned on the AGAC corpus [5] for fitting task. The NEN step, on the other hand, leverages existing tools such as PubTator and OGER +  + . The model’s performance is evaluated using precision, recall, and F1-score. Further details of the pipeline and its evaluation can be found in our previous work [7]. Finally, the annotated rice corpus encompasses 4195 GARE records from 32,229 abstracts and 56,368 full-text articles.

## Task definition

In this project, we utilize LLM for the extraction of GARE from the rice-related literature. To clarify the process, as illustrated in Fig. 1B, we adopt a prompt engineering strategy. Initially, raw sentences and task intructions are employed to query the LLM through the MoonShot API. Following this, we expect the LLM to generate GARE records. Ultimately, the generated results will be evaluated automatically.

There are still several challenges for LLM. Firstly, LLMs cannot utilize annotations from pipelines, e.g., NER and RE. This necessitates a deeper comprehension of sentence semantics to bypass the intermediate steps of the pipeline. Secondly, due to token limitations in the prompt, only a limited number of GARE examples can be provided, resulting in a few-shot learning problem. Thirdly, considering the rice context, there is an added emphasis on the need for LLMs to possess a broad knowledge background.

## Prompt engineering

During BLAH 8, we design and test various versions of prompts, ultimately arriving at the final version consisting of five sections, as outlined below. The complete prompt can be found in GitHub repositories.

- This section is designed to guide Kimi in understanding the concepts involved and the requirements of the task.
- Extraction rules: This component delineates specific extraction rules tailored for the task, such as ensuring each GARE record contains only one independent gene.
- Format requirement: Here, Kimi is instructed to present results in a predefined format, e.g., *Gene → Genetic Alteration → Trigger words → Rice Trait*, facilitating subsequent analysis and evaluation.
- Raw sentences: These sentences are provided to Kimi for GARE extraction, devoid of explicit annotations, thus prompting autonomous identification and extraction of relevant elements.
- Examples: This section presents three carefully selected cases from the annotated rice corpus, chosen for their distinctiveness and representativeness. These examples aim to enhance Kimi’s understanding of the task requirements by providing varied instances for reference.

### Experimental setup

In this project, we employed the web version of Kimi for prompt testing. For batch computations involving moonshot-v1-8 k, API version of Kimi was facilitated by the *openai* Python library. The code for making requests and usage instructions is available in the GitHub repository.

## Results

In this section, we perform automated evaluation and manual inspection and analyze specific instances of the generated results by the LLM.

### Automated evaluation

We manually curate 70 records from the annotated rice corpus as a golden dataset, including 50 positive samples with 89 GARE instances and 20 negative samples without GARE descriptions.

Using a lenient metric, we compare the generated GARE with the ground truth, considering overlap for all four elements as correct. For example, for the GARE “*OsZHD1 → overexpression → enhances → root growth*,” if the generated GARE is “*OsZHD1 gene → overexpression → significantly enhances → root growth*,” despite the additional words “*gene*” and “*significantly*,” we deemed it a correct prediction.

Table 1 presents the results of the AGAC-based pipeline alongside Kimi on this task. Kimi exhibits considerably higher precision (+ 0.14) relative to AGAC-based pipeline methods, while Kimi’s lower recall (− 0.11) results in an equivalent F1-score (− 0.007).

**Table 1:** Automated evaluation of rice GARE extraction

**Methods**	**Precision**	**Recall**	**F1-score**
AGAC-based pipeline [5]	0.5646	**0.6194**	**0.5907**
Kimi	**0.7010**	0.5000	0.5837

As mentioned above, the low accuracy of AGAC-based pipeline methods may stem from cascading errors or topic limitations, while their higher recall might be offset by increased false positives. In contrast, the result of LLM suggests that it has the potential to tackle these two challenges, albeit with a tightly constrained prompt that may lead to a reduced loss rate.

## Manual check

We conduct manual inspections on the 50 generated rice GAREs to identify errors and observe several common ones.

Firstly, errors in recognizing the boundaries of GARE elements are evident. “*TOM1 or HvTOM1*” is identified as a single gene, whereas it should have been split into two genes as per the benchmark dataset. Secondly, misidentification of genes and rice traits exists, such as the wrong labeling of the rice variety “*Bt rice T1C-19*” as a gene. Additionally, Kimi generates chains with missing traits, like identifying “*tryptamine*” in a sentence where it is not recognized as a separate rice trait but rather as a chemical compound.

Despite these errors, we note that Kimi naturally resolves the co-referencing task. For example, it correctly replaces “*either gene*” with specific genes, “*OsSTAR1*” and “*OsSTAR2*” in the sentence “*OsSTAR1 and OsSTAR2 function in Al tolerance in rice, and the disruption of either gene severely increases Al toxicity*.”

Furthermore, Kimi combines semantic meanings effectively to produce coherent results. For instance, it identifies a genetic alteration as a “*semi-dominant mutant*” in the sentence “*Introduction of the semi-dominant Bc6 mutant gene into wild-type rice significantly reduced the percentage of cellulose, causing brittle phenotypes. brittle phenotypes*,” which is challenging for traditional NER methods to articulate.

## Discussion and conclusion

This project presents an initial exploration into LLM-assisted bioregulatory event extraction. The evaluation results indicate that LLM demonstrates robust semantic comprehension and possesses a comprehensive knowledge base relevant to this task, surpassing conventional text mining pipelines. However, in specialized domains such as biology and agriculture, the superiority of LLM over traditional methods remains uncertain and varies. This uncertainty stems from multiple factors such as the obscurity of domain-specific terminology, the complexity of semantic intricacies, and the availability of relevant training samples for LLM. For instance, in biology, proprietary terms such as gene expression, transcription, and translation encompass complex biological associations that are difficult for LLMs to accurately comprehend. Further, gene expression can denote either gene activity or specific regulatory mechanisms, depending on the context. The scarcity of reports and the inaccessibility of literature in specific domains further constrain the capabilities of large language models.

We address several concerns related to further LLM applications.

First, there is a challenge in LLM-based knowledge curation in a low-resource domain. The accumulation of training texts is a common-sense solution to handle this challenge, and it benefits typical NLP tasks including NER, RE, and GARE extraction. For instance, within the medical field, particularly in the study of rare diseases, texts such as case reports and clinical trial data are utilized to compensate for the scarcity of medical literature. Furthermore, handling complicated tasks like NEN requires extensive development of controllable vocabulary or domain ontology and techniques in processing and understanding long contexts.

Second, the computational cost of the LLM API usage may hinder its widespread adoption in large-scale applications. Nevertheless, a possible trend toward open-source solutions is expected to mitigate this issue over time.

Third, a theoretical assessment metric for LLM-based prompt design is lacking. To our greatest knowledge, the main assessment of the prompt is empirically initiated, which mainly focuses on indirect evaluation of the corresponding NLP task, instead of the prompt itself. The development of a theoretical metric to for prompt optimization remains an unsolved problem, and the solution of this problem sheds light on the deeper evolvement of LLM applications.

## Data Availability

No datasets were generated or analysed during the current study.
